# Metal–Organic Framework-Based Sustainable Nanocatalysts for CO Oxidation

**DOI:** 10.3390/nano10010165

**Published:** 2020-01-17

**Authors:** Luis A. Lozano, Betina M. C. Faroldi, María A. Ulla, Juan M. Zamaro

**Affiliations:** Instituto de Investigaciones en Catálisis y Petroquímica, INCAPE (FIQ, UNL, CONICET), Santiago del Estero 2829 (3000), Santa Fe, Argentina; llozano@fiq.unl.edu.ar (L.A.L.); bfaroldi@gmail.com (B.M.C.F.); mulla@fiq.unl.edu.ar (M.A.U.)

**Keywords:** UiO-66, copper, iron, cobalt, nanocatalyst, CO oxidation, COProx

## Abstract

The development of new catalytic nanomaterials following sustainability criteria both in their composition and in their synthesis process is a topic of great current interest. The purpose of this work was to investigate the preparation of nanocatalysts derived from the zirconium metal–organic framework UiO-66 obtained under friendly conditions and supporting dispersed species of non-noble transition elements such as Cu, Co, and Fe, incorporated through a simple incipient wetness impregnation technique. The physicochemical properties of the synthesized solids were studied through several characterization techniques and then they were investigated in reactions of relevance for environmental pollution control, such as the oxidation of carbon monoxide in air and in hydrogen-rich streams (COProx). By controlling the atmospheres and pretreatment temperatures, it was possible to obtain active catalysts for the reactions under study, consisting of Cu-based UiO-66-, bimetallic CuCo–UiO-66-, and CuFe–UiO-6-derived materials. These solids represent new alternatives of nanostructured catalysts based on highly dispersed non-noble active metals.

## 1. Introduction

The use of metal–organic frameworks (MOFs) as host matrices to disperse metal nanoparticles is a topic of great interest in the ongoing research for new nanocatalysts. MOFs have the advantage of presenting a variety of transition metals and a wide range of organic ligands in their composition, which makes them attractive for applications in catalysis [1,2]. There are several alternatives to obtain catalytic functionality in the structure of MOFs. One is based on the coordination around metal centers or structural defects that are not committed to the material framework, which can act as active sites [3]. Another possibility is to take advantage of the ligand chemistry since terephthalates or tricarboxylates to which acidic or basic functional groups can be added are usually used [4]. Moreover, the large internal volume available in these materials can be used to host active species [5]. In addition, in recent years, the use of MOFs as templates, which according to their construction units can generate nanostructured metal/metal oxide systems [6], became a consolidated strategy. For example, Sun et al. [7] reported a number of catalysts obtained with this concept, such as Co_3_O_4_ materials from Co-based MOFs or α-Fe_2_O_3_ and Fe_3_O_4_ nanomaterials from Fe-MIL-88B. CuO and CuO–CeO_2_ nanoparticle-based catalysts derived from the MOF HKUST-1 with high catalytic activity were also reported [8].

On the other hand, the catalytic oxidation of carbon monoxide in gaseous streams is a reaction of great environmental relevance. Carbon monoxide is one of the main pollutants of indoor and industrial environments because, due to its high affinity for hemoglobin, it is extremely toxic to living beings [9] and, therefore, numerous studies are currently being conducted for their catalytic removal [10]. Moreover, the elimination of this gas in concentrated hydrogen streams (COProx) has significance in the field of renewable energy, as it is one of the most accepted alternatives to carry out the final purification of H_2_ to be used in fuel cells [11]. For the CO oxidation, numerous catalytic formulations were tested to date, and those composed of supported oxides represent one of the most promising alternatives [12]. For example, catalysts based on supported particles of copper oxides, cobalt oxides, and iron oxides showed good performance [13,14,15]. These types of solids avoid the use of noble metals traditionally used in this reaction such as Pt, Pd, or Au [12], some of which were also supported in MOFs [16,17]. These elements have a limited abundance and involve much higher costs; thus, efforts are being made for the development of catalysts based on non-noble metals. Currently, it is considered that the use of precious metals is not sustainable compared to earth-abundant metals which are available in orders-of-magnitude higher quantities [18]. Of special interest are the metals of the first transition period, such as Cu, Co, and Fe, since they are not only less expensive but also less toxic compared to those of the second and third period [19]. Since MOFs have high specific surface areas, they represent a new support alternative for the efficient and low-cost obtainment of catalysts based on dispersed non-noble metal species. In this scenario, UiO-66 is an attractive structure for this purpose since it is a microporous zirconium terephthalate forming a three-dimensional arrangement with high specific surface area and good thermal, mechanical, and chemical stability [20]. In addition, it requires low-cost precursors for its synthesis and can be obtained under fairly sustainable conditions. Very recently, active catalysts based on atomically dispersed ionic Cu species on UiO-66 and hybrid nanostructures of CuO nanocrystals encapsulated in UiO-66 crystals were reported [21,22]. In addition, with the concept of a matrix, CuO/CeO_2_ active catalysts derived from Ce–UiO-66 were obtained, as well as CuCe/ZrO_2_ catalysts derived from metal-impregnated UiO-66 [23,24].

In this context, the present work proposes to systematically analyze the use of UiO-66 synthesized through a sustainable protocol [25] as a dispersion matrix of Cu, Co, and Fe species to obtain new nanoparticle structures with potential use in catalysis. In addition, the incorporation of these metallic species is proposed by simple procedures traditionally used to prepare supported catalysts, such as the incipient wetness impregnation with precursors and subsequent thermal decomposition. The physicochemical properties of the obtained nanomaterials were tested in the gas-phase oxidation of carbon monoxide in air streams and in the CO preferential oxidation in hydrogen-rich streams (COProx).

## 2. Materials and Methods

### 2.1. Synthesis of UiO-66

Benzenedicarboxylic acid (BDC, Aldrich, 98.0% purity, St. Louis, MO, USA), ZrCl_4_ (Zr, Aldrich, 98.0% purity, Darmstadt, Germany), and acetone (Cicarelli, 99.0% purity, San Lorenzo, Argentina) were used without further purification, and UiO-66 synthesis was performed employing a sustainable protocol reported elsewhere [25]. Briefly, the procedure consisted of mixing the two solid reactants together with the solvent in the molar proportions BDC:ZrCl_4_:solvent = 1:1:1622. After obtaining the homogeneous mixture, it was placed under solvothermal treatment at 80 °C for 24 h. At the end of the treatments, the solids were recovered by centrifugation (10,000 rpm, 10 min), washed twice with ethanol, and finally dried at 80 °C overnight.

### 2.2. Incorporation of Metallic Species

Cu(NO_3_)_2_∙3H_2_O (Aldrich, 98.0–103% purity, St. Louis, MO, USA), Co(NO_3_)_2_∙6H_2_O (Alfa Aesar, 98.5% purity, Tewksbury, MA, USA), and Fe(NO_3_)_3_∙9H_2_O (Aldrich, ≥99.99%, purity, St. Louis, MO, USA) were employed as metal precursors. For their incorporation to MOF, incipient wetness impregnation was used as described in S1 (Supplementary Materials). The nomenclature employed was the following: First the incorporated metal, then its load in wt.% with respect to the catalyst total mass, and finally the support which was MOF (M) or its degradation product (Zr), i.e., Cu_10_/M or Cu_16_Fe_7_/Zr.

### 2.3. Catalyst Characterization

The crystalline structure of the materials was analyzed trough X-ray diffraction (XRD) with a Shimadzu XD-D1 instrument (Shimadzu Corp., Kyoto, Japan, CuKα radiation, λ = 1.5418 Å, 2 °C∙min^−1^, 30 mV, 40 mA, 2θ = 5° to 65°). In order to analyze the thermal evolution of the MOF and precursor, thermogravimetric analysis (TGA) and single differential thermal analysis (SDTA) were conducted with a Mettler Toledo STARe (version 4.1, Bristol, UK) TGA/SDTA 851e module from 25 to 800 °C at 10 °C∙min^−1^ in air or nitrogen flow (50 mL∙min^−1^, standard temperature and pressure (STP)). Laser Raman spectroscopy (LRS) was performed using a LabRam spectrometer (Horiba-Jobin-Yvon, Stanmore, UK) coupled to an Olympus confocal microscope (Olympus Corp., Shinjuku, Tokyo, Japan) equipped with a charge-coupled device (CCD) detector cooled to about 200 K. The excitation wavelength was 532 nm (Spectra Physics argon-ion laser), and the laser power was set at 30 mW. Transmission electron microscopy (TEM) images of the synthesized UiO-66 crystals and metal-based nanocatalysts were acquired using a JEOL 2100 Plus microscope (JEOL Ltd., Tokyo, Japan) equipped with an energy dispersive X-ray (EDX) detector (JEOL Ltd.) and a scanning transmission electron microscope (STEM) (JEOL Ltd.). The samples were milled and suspended in ethanol by ultrasonic treatment, and a drop of the fine suspension was placed on a carbon-coated nickel grid to be loaded into the microscope.

### 2.4. Catalytic Evaluations

The samples were evaluated in a glass tubular reactor connected to a continuous flow system equipped with mass flow controllers (Brooks 4800, Brooks Instrument, Hatfield, PA, USA) and heated with a furnace controlled with a proportional–integral–derivative (PID) system. Before each evaluation, the reactor was heated at different temperatures (200–650 °C) in He or Air flow (30 mL∙min^−1^) according to the pretreatment required by the sample before its test and maintaining such a temperature for 60 or 120 min. Then, the catalytic tests were performed with a mixture of molar composition 1% CO, 2% O_2_ (employing synthetic air) in He balance, maintaining a total flow of 30 mL∙min^−1^ with an initial mass of the solid of 70 mg. Some tests were also performed by adding 50% of H_2_ in the reaction stream (COProx), at the same total flow. The catalytic measurements were taken after stabilizing the reactor at different temperatures for 8 min. The CO conversions were determined with an on-line Shimadzu GC-2014 chromatograph (Shimadzu Corp., Kyoto, Japan) equipped with a thermal conductivity detector (TCD) and a 5-Å molecular sieve packed bed column. CO conversions (XCO were calculated as follows:

XCO (%) = ([CO]^0^ − [CO])/[CO]^0^ × 100,

where [CO]^0^ and [CO] are inlet and outlet gas concentrations (ppm), respectively. Moreover, for COProx, the selectivity of O_2_ to CO_2_ (S) was calculated as follows [12]:

S (%) = XCO (%)/(λ × (([O_2_]^0^ − [O_2_])/[O_2_]^0^)),

where [O_2_]^0^ and [O_2_] are inlet and outlet oxygen concentrations (ppm), respectively. In our case, the value of the factor λ (excess oxygen in the reaction) was four.

## 3. Results and Discussion

### 3.1. Dispersion of Copper Species in UiO-66

#### 3.1.1. Study of the Precursor Decomposition

**Figure 1:**
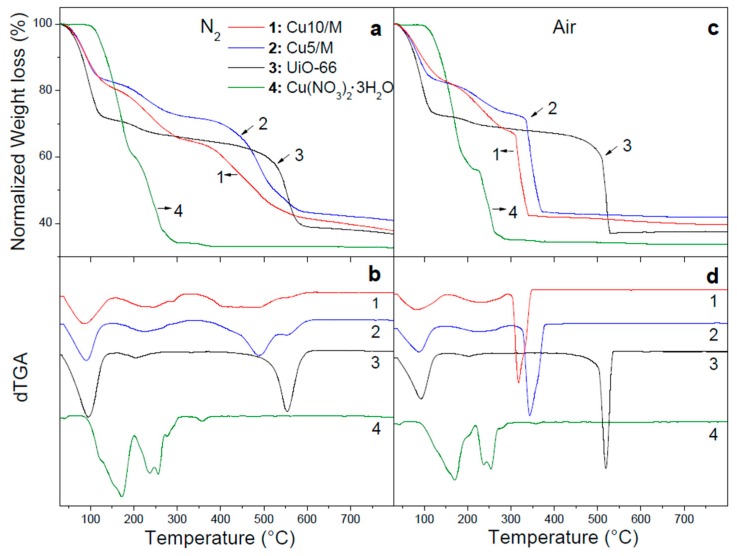
Thermogravimetric analysis (TGA) of UiO-66 (M), Cu/M, and copper precursor: (**a**) N_2_ atmosphere; (**c**) air atmosphere. Corresponding derivative TGA (dTGA) curves: (**b**) N_2_ atmosphere; (**d**) air atmosphere.

The obtained MOF corresponded to a crystalline and pure phase of UiO-66 since all its diffraction signals were observed (Figure S1, Supplementary Materials); the most important ones were identified at 2θ = 7.38°, 8.52°, and 25.75°, corresponding to the (111), (200), and (600) planes, respectively [20]. All these signals matched those indexed for this MOF from its crystallographic data (CCDC 733458) and no impurities were detected, such as benzenedicarboxylic acid. Afterward, the compatibility between the decomposition temperatures of the metal precursors and the MOF was analyzed by TGA, both in an inert and in air atmosphere. During the impregnation process, the transition metals were incorporated as nitrate salts onto the UiO-66 surface. Afterward, the nitrate ion decomposition was required to obtain the metal oxides. In this context, it is necessary to analyze the atmosphere and temperature condition in which this decomposition was done, maintaining the MOF structure. Figure 1a, profile 3, presents the TGA of UiO-66 in inert gas, and Figure 1b, profile 3, depicts its corresponding derivative TGA (dTGA). Typical evolutions were observed, with an initial mass loss up to 130 °C due to physisorbed water and/or residual solvent of synthesis. Then, another small mass loss from 180 °C due to the dehydroxylation of the inorganic cluster of the MOF from Zr_6_O_4_(OH)_4_ to Zr_6_O_6_. Finally, the thermal decomposition of the ligands was observed, which was similar to the one corresponding to this MOF synthesized under conventional conditions [26], with a maximum decomposition rate (T^max^) at 555 °C (Figure 1(b3) and Table 1). Instead, under air atmosphere, the TGA (Figure 1(c3)) and dTGA profiles (Figure 1(d3)) showed a somewhat lower stability with a T^max^ of 520 °C (Table 1). This evolution was more exothermic compared to the previous one as could be seen in the SDTA (Figure S2, Supplementary Materials). Meanwhile, the copper precursor showed a complete decomposition at 290 °C or 265 °C in inert gas (Figure 1(a4)) or air (Figure 1(b4)), respectively. The degradation temperature window of the MOF and the precursor suggested the possibility of obtaining dispersed copper species by applying heat treatments.

The MOF impregnated with 5 wt.% copper (Cu_5_/M) showed a marked decrease in the structural stability of the framework (Figure 1a,b), which was magnified with a higher copper amount (Cu_10_/M). The T^max^ was reduced from 555 °C to 490 °C and 460 °C for fresh UiO-66, Cu_5_/M, and Cu_10_/M, respectively (Table 1). The same effect was observed in air but with a greater destabilization of the framework. This phenomenon is attributed to the copper species formed after the precursor decomposition, which catalyzed the oxidation of the organic part of the MOF, accelerating its structural collapse. However, a small temperature gap persisted in which it would be possible to decompose the said precursor before the MOF collapsed. The SDTA of Cu/M in both atmospheres (Figure S2, Supplementary Materials) showed only an endothermic process due to the evacuation of host molecules that ended at 160 °C, and an exothermic one near 300 °C due to MOF collapse. From the previous results, pretreatments of Cu_10_/M in inert and combinations with air were carried out at different temperatures prior to carrying out its catalytic test in order to get insight into the catalytic performances and structural stabilities after the different pretreatments.

#### 3.1.2. Copper-Based UiO-66 Catalysts

**Figure 2:**
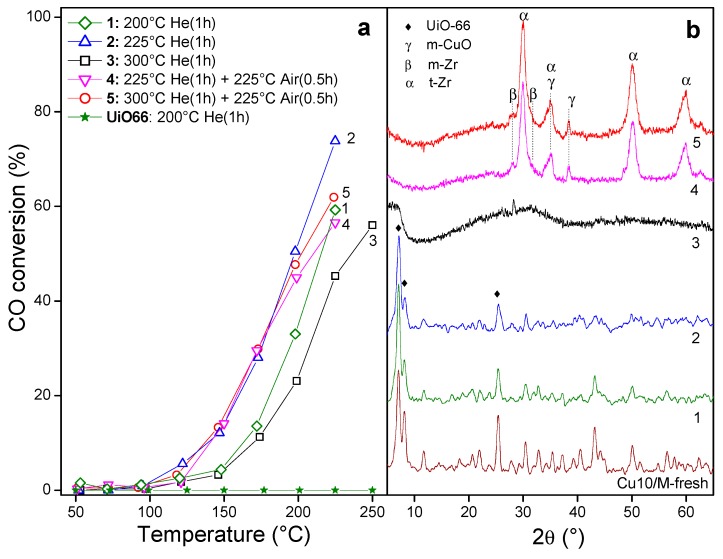
Cu/UiO-66 solids (Cu/M): (**a**) catalytic evaluation in the CO oxidation; (**b**) X-ray diffraction (XRD) patterns after the reaction.

In Figure 2a it can be observed that UiO-66 by itself presented no activity in the oxidation of CO. On the other hand, Cu_10_/M treated for 1 h at 200 °C in He (1) presented activity, reaching a maximum conversion of 60% at the said temperature, while a treatment at 225 °C (2) yielded an improvement, reaching conversions of 75%. Meanwhile, a pretreatment at 300 °C impaired activity, as observed in solid (3). The diffractograms of these evaluated samples (Figure 2b) indicate that samples (1) and (2) maintained the MOF structure. Nevertheless, a degradation of the MOF took place in sample (3). The absence of definite signals of copper species in all the diffractograms should be highlighted since it indicates their high dispersion. Accordingly, in solids (1) and (2), the catalytic activity was due to copper species highly dispersed in the MOF structure, while, in sample (3), those species were dispersed in an amorphous solid.

The change in the pretreatment atmosphere was analyzed in terms of the activity of Cu_10_/M by combining the pretreatment in inert atmosphere followed by a brief exposure to air for 0.5 h (sample (4)). The as-treated solid was also active (Figure 2a) even though the treatment promoted the total degradation of the MOF (Figure 2b), favoring its evolution to a crystalline phase of tetragonal zirconia (t-Zr), (JCPDS 17-923). The decomposition of the hydrated nitrate salts in the presence of air can generate various oxidizing agents such as HNO_3_ and NO_2_, which, added to the treatment in oxygen, can give rise to a hyperoxidizing atmosphere and accelerate the transformation of the MOF into zirconia. This evolution is in agreement with what was reported for the decomposition of UiO-66 in air [27]. A high dispersion of cupric oxide was observed in these solids, characterized by weak signals at 2θ = 35.5° (masked by a signal of t-Zr) and 38.5°, corresponding to the (11−1) and (111) planes of a CuO monoclinic phase (JCPDS 48-1548), respectively.

**Figure 3:**
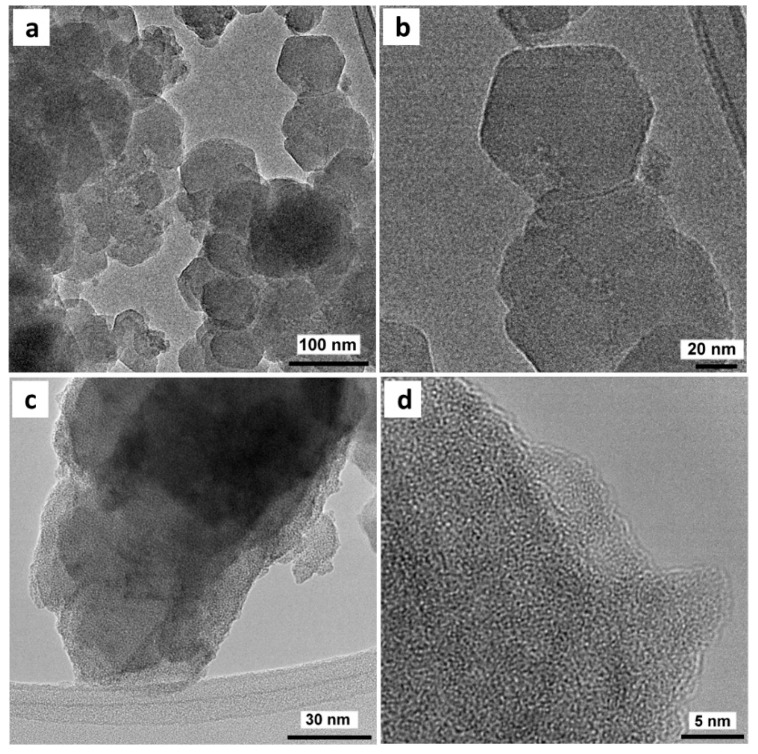
TEM images: (**a**) as-synthesized UiO-66 crystals; (**b**) close view of UiO-66; (**c**) Cu/UiO-66 catalyst with He treatment at 225 °C; (**d**) close view of activated (He treated) Cu/UiO-66.

The TEM image of the synthesized MOF shows nanometric crystals with sizes ranging from 30–100 nm which formed globular aggregates (Figure 3a), making it possible to distinguish the facets of the individual crystals with a polyhedral morphology (Figure 3b) similar to that reported for this MOF but obtained under conventional conditions [20]. Meanwhile, when the said crystals were functionalized with copper following the sequence of impregnation and heat treatment in He to obtain Cu/UiO-66, the porous structure of the MOF was maintained (Figure 3c). The high-resolution (HR) TEM image showed the characteristic aspect of a porous material but no particles could be distinguished inside the MOF porosity (Figure 3d). This highlights the small size of these copper species dispersed in the MOF, consistent with the XRD results.

In brief, a pretreatment of Cu_10_/M in He for 1 h at 225 °C allowed obtaining an active catalyst in the CO oxidation, based on copper species with high dispersion in the MOF structure which were preserved after the tests in reaction. This nanocatalyst represents a new alternative not only for this reaction but also for other reactions demanding a high dispersion of active copper phases and led at relatively low temperatures (<225 °C). These reactions could be, for example, the reduction of C–C multiple bonds and carbonyl, the hydroxylation of benzene, the reduction of aromatic nitrocompounds or NO_x_ [28], or the synthesis of methanol from CO and H_2_ [29].

### 3.2. Derived Cu/UiO-66 Catalysts

#### 3.2.1. Monitoring of the Thermal Transformation of Cu/MOF

**Figure 4:**
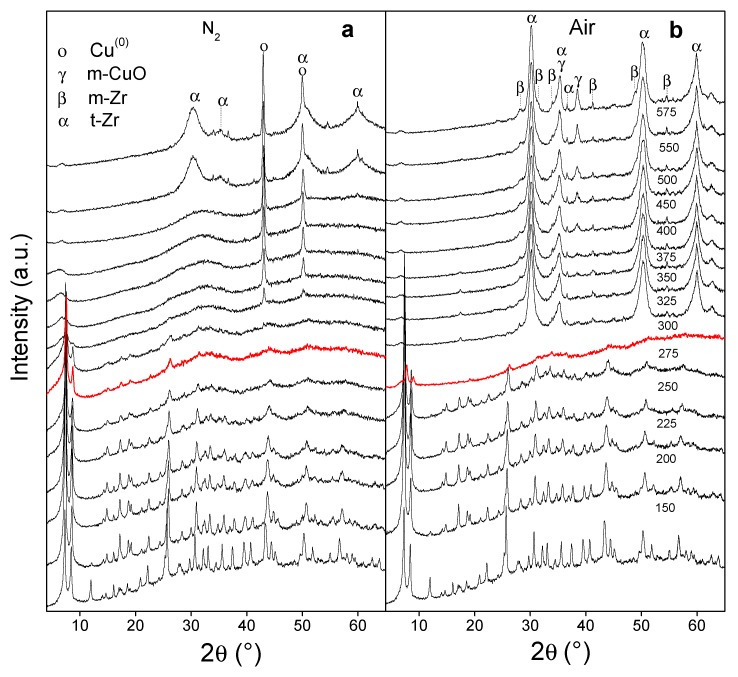
Analyses of X-ray diffraction at programmed temperature (T-XRD) with the Cu/M sample: (**a**) in nitrogen; (**b**) in air.

Given the structural changes observed after the thermal pretreatments, the transformation of Cu/M was analyzed through temperature-programmed X-ray diffraction (T-XRD) both in an inert atmosphere and in air. In the first case, from 250 °C, the MOF underwent a reduction in crystallinity (Figure 4a), totally losing itself at 325 °C (red curve). Then, the solid persisted as an amorphous material in which signals at 43.3° and 50.1° emerged (the latter masked with a t-Zr signal), which corresponded to the (111) and (200) planes of a cubic Cu^0^ phase, respectively (JCPDS 4-836). At higher temperatures, these species increased the crystallinity, while the support evolved into a tetragonal zirconia (t-Zr) of low crystallinity. This is consistent with what was discussed above. On the other hand, heat treatments in air showed that the structure of the MOF was more unstable (Figure 4b) losing the crystallinity at 275 °C (red curve). In addition, it quickly transformed into a t-Zr system with a high dispersion of copper oxide species. The stable formation of a t-Zr phase from this MOF was attributed to the initial transformation of the small zirconia nuclei from inorganic nodes, which have a low surface energy and facilitate the evolution toward a tetragonal phase instead of a monoclinic (m-Zr) [27]. From about 400 °C a small contribution of m-Zr was detected, characterized by strong signals at 2θ = 27.9°, 31.2°, 34.1°, 40.7°, and 49.1°, corresponding to the (−111), (111), (200), (−112), and (220) planes, respectively (JCPDS 37-1484). An increase in the proportion of the monoclinic phase in copper-doped zirconia was attributed to copper inclusion in the ZrO_2_ network, which increased the size of the crystallites, causing a growth in the free surface energy and, thus, promoting the evolution toward m-ZrO_2_ [30]. This could be, in our case, due to a migration of part of the copper to the zirconia phase in formation during the heat treatment in air.

By T-XRD, it was shown that thermal pretreatments of the Cu/M solid in an inert atmosphere caused a delayed degradation of the MOF toward an amorphous solid in which Cu^0^ species evolved. Meanwhile, the MOF degradation was accelerated in air with a fast growth of t-Zr phase with a small contribution of m-Zr and with highly dispersed CuO species. Given the potential of solids derived from Cu/M, their catalytic behavior was analyzed.

#### 3.2.2. Catalytic Behavior of Derived Cu/UiO-66 Catalysts

**Figure 5:**
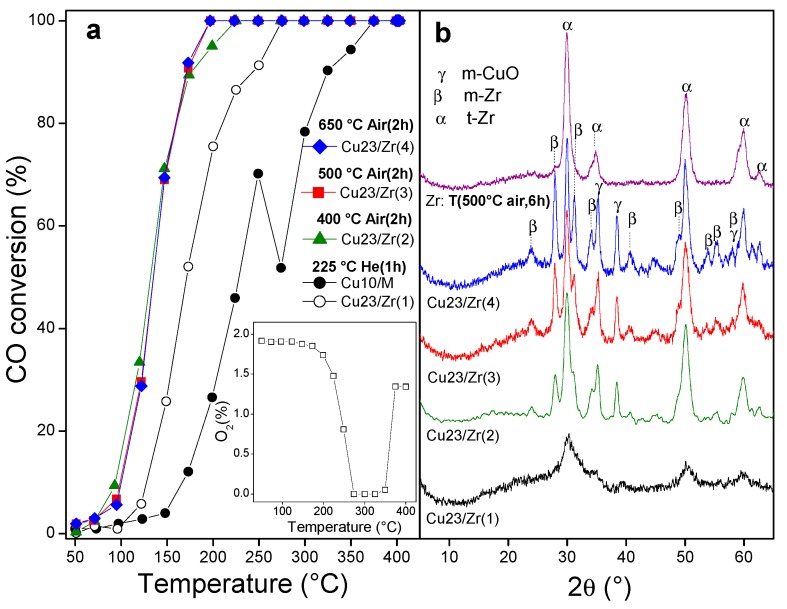
Cu/UiO-66-derived solids: (**a**) catalytic evaluation; (**b**) XRD patterns after reaction.

Cu_10_/M was pretreated in situ in He flow at 225 °C for 1 h, and its catalytic curve showed an inflection in the profile starting at 250 °C (Figure 5a) due to a fall in the CO conversion. This was caused by a smaller availability of oxygen for the reaction (as observed in the insert in Figure 5a), which was consumed in the MOF degradation. Hence, 250 °C is the maximum temperature at which Cu_10_/M maintained its structure under reaction conditions. From 375 °C the oxygen was recovered, and the catalyst was taken up to 400 °C for 1 h in reaction, maintaining conversions of 100%; later, the catalyst was cooled and evaluated again. In this case, Cu_23_/Zr (1), a marked activation was observed (Figure 5a) due to the evolution of the solid to the system of copper oxide dispersed in a developing t-Zr phase (Figure 5b). Since the zirconia mass in the derived solid was around 37.4 wt.% with respect to the initial mass of the MOF, the proportion of copper in these systems was 23 wt.%. It is noticeable that, with this high load, the copper species were highly dispersed in the t-Zr support. Given the good catalytic performance of these solids and taking into account the studies of the transformation of a Cu/M solid into Cu/Zr, a pretreatment of Cu_10_/M in air was performed at 400 °C for 2 h. In this case, Cu_23_/Zr (2), a remarkable shift of the catalytic curves was observed, reaching total conversion at 225 °C (Figure 5a) without extra oxygen consumption due to the presence of a stabilized phase of CuO/ZrO_2_ (Figure 5b) with a contribution of a m-Zr phase. Compared with classical CuO/ZrO_2_ catalysts obtained via other techniques such as sol–gel [31] or urea combustion [32], the use of the MOF as a template allowed minimizing the generation of bulk CuO of low interaction with zirconia, which would generate a lower catalytic activity. In contrast to the MOF-derived zirconia (Zr in Figure 5b), the Cu_23_/Zr (2) solid showed a contribution of the m-Zr phase. When Cu_10_/M was pretreated in air at 500 °C, Cu_23_/Zr (3), a slight catalytic improvement was observed with respect to the former case, reaching total conversion at 175 °C.

However, the contribution of m-Zr in this sample was more evident, which could be related to its better catalytic behavior. In this sense, it was demonstrated that the adsorption capacity of CO in m-Zr supports was higher than in t-Zr, which can be explained by a higher Lewis acidity (Zr^4+^), as well as a higher Lewis basicity (O^2−^), on the surface of the m-Zr solid [33]. Finally, a pretreatment at 650 °C in air, Cu_23_/Zr (4), did not improve the conversion (Figure 5a), even though it favored the development of the monoclinic phase, probably due to a sintering of the CuO species (Figure 5b).

### 3.3. Derived Cobalt and Iron-Based UiO-66 Catalysts

Other non-noble metals of interest that have activity in the CO oxidation reaction are cobalt and iron [13], added to the fact that the latter is a very low-cost metal with high abundance. The decomposition temperature of cobalt and iron nitrate precursors in air was far from the limit of MOF stability (Table 2, Figure S3, Supplementary Materials) while the incorporation of 10 wt.% Co or Fe in the MOF (Co_10_/M, Fe_10_/M) decreased the framework stability due to the formed oxides, although the shift was lower than that of Cu_10_/M. The order of structural stability was as follows: Co/M > Fe/M > Cu/M. The SDTA profiles in air (Figure S3, Supplementary Materials) were very similar to that of Cu_10_/M, with an endothermic peak due to the evaporation of host molecules and an exothermic one due to structural collapse. Taking into account the similar structure stability of Cu_10_/M under either He or air atmosphere at temperatures lower than 275 °C, Co_10_/M and Fe_10_/M solids were pretreated at 250 °C in air, and their catalytic behavior was analyzed.

For the Co_10_/M solid (Figure S4a, Supplementary Materials), from 200 °C onward, conversion increased proportionally with temperature, and, when it was over 325 °C, both a conversion fall and an abrupt consumption of oxygen were produced due to the MOF degradation. The activity evolved until total conversion but at a higher temperature than the Cu_10_/M solid, previously analyzed. Subsequently, this solid was taken to 400 °C and was kept 1 h under reaction. When evaluated again, an improvement in activity was observed, Co_23_/Zr (1). The catalyst consisted of a t-Zr phase evolving with a high dispersion of cobalt species due to the absence of characteristic signals of their oxides (Figure S4b, Supplementary Materials). Since it was observed that an improvement in ZrO_2_ crystallinity favored the activity, a pretreatment of the Co_10_/M sample was performed in air but at 400 °C for 2 h. This effectively improved the catalytic performance (Figure S4a, Supplementary Materials) due to the formation of a stabilized phase of Co_3_O_4_ in a t-Zr of high crystallinity (Figure S4b, Supplementary Materials). This is in agreement with what was reported regarding the formation of this cubic spinel (JPDS 43-1003) on conventional ZrO_2_ supports [15].

On the other hand, Fe_10_/M showed less activity (Figure S4a, Supplementary Materials), even over 300 °C when the MOF decomposed. After 1 h in reaction at 400 °C, the system was evaluated again, and an improvement was observed even though total conversion was not reached in the temperature range analyzed. This solid consisted of an incipiently formed t-Zr with a high dispersion of iron species (Figure S4b, Supplementary Materials). In this case, a remarkable catalytic improvement was also observed when pretreating at 400 °C in air. This solid consisted of a highly crystalline t-Zr with a small contribution of m-Zr which dispersed a rhombohedral hematite phase (α-Fe_2_O_3_). The previous confinement of the iron precursor in the pores of UiO-66 facilitated, after degradation, the generation of small Fe_2_O_3_ crystals that were quite active in CO oxidation, as already observed for iron oxide crystals [34].

The MOF degradation under reaction conditions of CO oxidation started at 325, 300, and 250 °C for Co_10_/M, Fe_10_/M, and Cu_10_/M respectively. Although the thermal stability of the latter was slightly lower, its CO conversion at 250 °C was significantly higher (70%) than that of the other two samples (17% and 5%). The best catalytic performance corresponded to the Cu-based sample after the degradation of the MOF structure in air at 400 °C for 2 h (Cu_23_/Zr), which reached 100% CO conversion at 225 °C. At that temperature, the conversion for the Co_23_/Zr solid was 45%, while, for the Fe_23_/Zr solid, it was only 8%.

### 3.4. Bimetallic CuCo/UiO-66- and CuFe/UiO-66-Derived Nanocatalysts

#### 3.4.1. CO Oxidation

**Figure 6:**
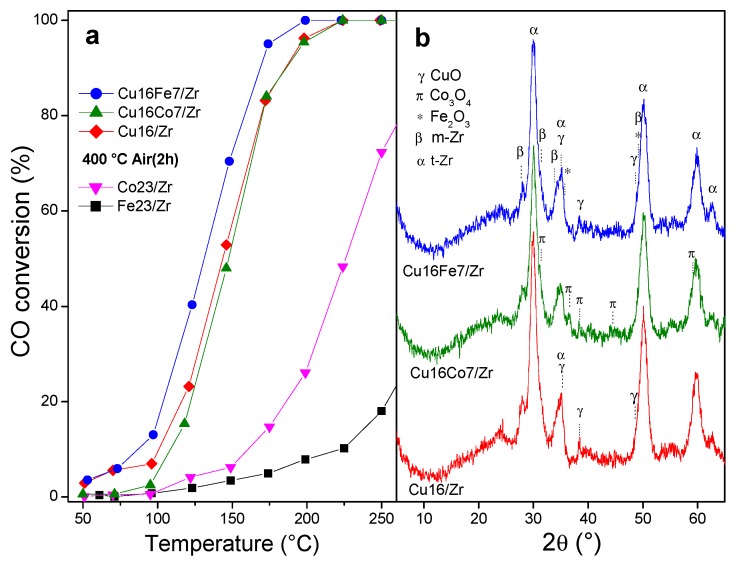
CuCo/Zr and CuFe/Zr nanocatalysts: (**a**) catalytic behavior; (**b**) XRD patterns after reaction.

It was reported that mixed cobalt and copper oxides [35], as well as copper–iron mixed oxides synthesized by low-temperature co-precipitation methods [33,36,37], have synergistic effects on the oxidation of CO; therefore, bimetallic systems were prepared incorporating these metals into UiO-66. The solids obtained by successive impregnation were analyzed, firstly by incorporating the copper precursor followed by their thermal treatment (500 °C, air, 2 h); subsequently, a cobalt or iron precursor was added, followed by a final calcination step in air (400 °C, 2 h) to obtain Cu_16_Co_7_/Zr and Cu_16_Fe_7_/Zr systems. The Cu_16_/Zr, Cu_16_Co_7_/Zr, and Cu_16_Fe_7_/Zr samples exhibited well-developed t-Zr phases with a small contribution of m-Zr (promoted by the presence of copper as discussed above), adding to a CuO phase with high dispersion (Figure 6). Additionally, a Co_3_O_4_ phase was observed in the solid containing cobalt, while, in the iron-containing bimetallic solid, no iron oxide phases were detected. This accounts for the high dispersion of the FeO phases in this solid. The catalytic assays showed that, among these catalysts, an improvement in the activity of Cu_16_Fe_7_/Zr was found (Figure 6a). This was due to both the initial presence of a very small proportion of m-Zr phase before the incorporation of iron and the subsequent development of a Fe–Cu synergy among these species due to their intimate contact, favored by the high dispersion achieved by these oxides in the solid, as shown by their XRD patterns.

**Figure 7:**
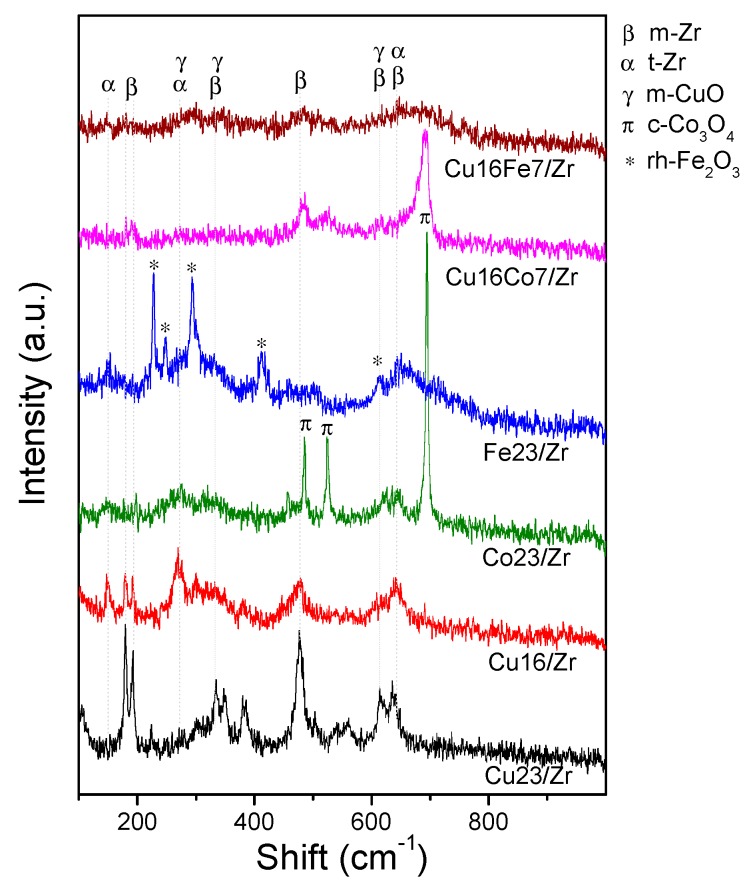
Laser Raman spectroscopy (LRS) spectra of the mono and bimetallic solids after the CO oxidation reaction.

The catalytically evaluated nanomaterials were analyzed by laser Raman spectroscopy (LRS). These spectra are shown in Figure 7, and the respective spectra of the used monometallic samples are included for comparison. The vibrational signals observed in all of these spectra are consistent with the crystalline phases identified by XRD. The vibrations of the monoclinic ZrO_2_ (m-Zr) and tetragonal ZrO_2_ (t-Zr) phases were present in the Cu_16_/Zr spectrum (Figure 7), proving the existence of both zirconia phases. The characteristic narrow vibration signals of m-Zr were at 179, 192, 335, 347, 385, 476, 614, and 635 cm^−1^, with that at 476 cm^−1^ being the strongest one [15], while the typical broad signals of t-Zr were at 145, 275, 310, 460, and 650 cm^−1^ [27]. In the case of the sample with higher copper content, Cu_23_/Zr, the monoclinic phase was clearly identified due to its narrow vibration signals (Figure 7). The vibrations of CuO at 280, 335, and 615 cm^−1^ [8] overlapped with those of the zirconia, thus hindering their identification. The spectrum of Co_23_/Zr pointed out the existence of a t-ZrO_2_ phase and a Co_3_O_4_ spinel (485, 523, and 687 cm^−1^) [15], in clear agreement with the XRD results. The absence of m-ZrO_2_ was evident, inferring that the UiO-66 degradation in the presence of cobalt hampered the formation of this monoclinic phase. The same outcome was obtained when iron was the impregnated metal. The Fe_23_/Zr spectrum (Figure 7) revealed the presence of just t-ZrO_2_ and α-Fe_2_O_3_ (226, 246, 293, 411, and 610 cm^−1^ [36]), and no signals of m-ZrO_2_ were identified.

In the spectra of the bimetallic catalysts, it could be observed that the zirconia signals were mainly associated with t-Zr (Figure 7). In the Cu_16_Fe_7_/Zr sample, a high dispersion of iron and copper oxides was achieved given the absence of defined signals of these phases, in line with what was observed by XRD and also confirming the absence of agglomerates after the reaction. This shows that the addition of Fe to the Cu/Zr system generated highly dispersed and stable iron species, since they were kept in that situation in the solid after reaction (Figure 7). This is in contrast with the higher sintering reached in the monometallic Fe/Zr system after reaction. On the other hand, in the spectrum of sample Cu_16_Co_7_/Zr, the signals of a developed Co_3_O_4_ spinel were dominant (Figure 7), in agreement with XRD observations, showing again that the cobalt species were segregated forming big crystals at the catalyst surface.

**Figure 8:**
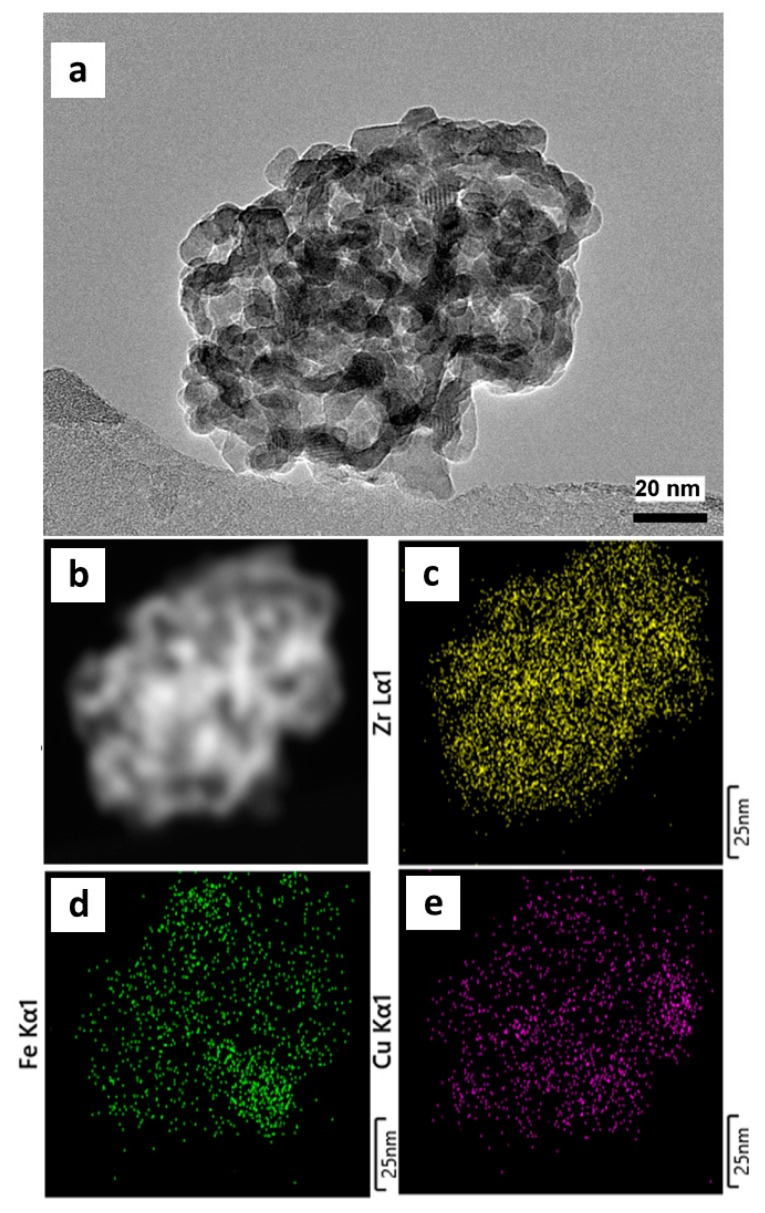
TEM images of Cu_16_Fe_7_/Zr catalyst: (**a**) selected area for the energy-dispersive X-ray (EDX) mapping in bright field; (**b**) selected area for the EDX mapping in dark field. Elementary mapping: (**c**) zirconium (yellow); (**d**) iron (green); (**e**) copper (magenta).

The bimetallic sample Cu_16_Fe_7_/Zr exhibited a nanoparticle system in intimate contact (Figure 8a), confirming the results of XRD and LRS discussed above, which corresponded to small domains of zirconia phases and oxides of copper and iron. The different crystalline planes of these phases in the individual crystals can be observed (Figure 8a). Figure 8b shows the analysis of the same particle in dark-field mode and its nature of aggregated nanoparticles was also highlighted. The elementary mapping performed in STEM mode showed a homogeneous distribution of the zirconium (yellow, Figure 8c), iron (green, Figure 8d), and copper (magenta, Figure 8e) phases in the nanoparticle aggregates, confirming the high dispersion and intimate contact between these nano-oxides. The characterizations performed by XRD, LRS, and TEM demonstrated the small particle size reached by the phases of these oxides in intimate contact with each other, explaining the catalytic synergy in this material, as shown in Figure 6.

#### 3.4.2. CO Oxidation in Hydrogen-Rich Stream (COProx)

**Figure 9:**
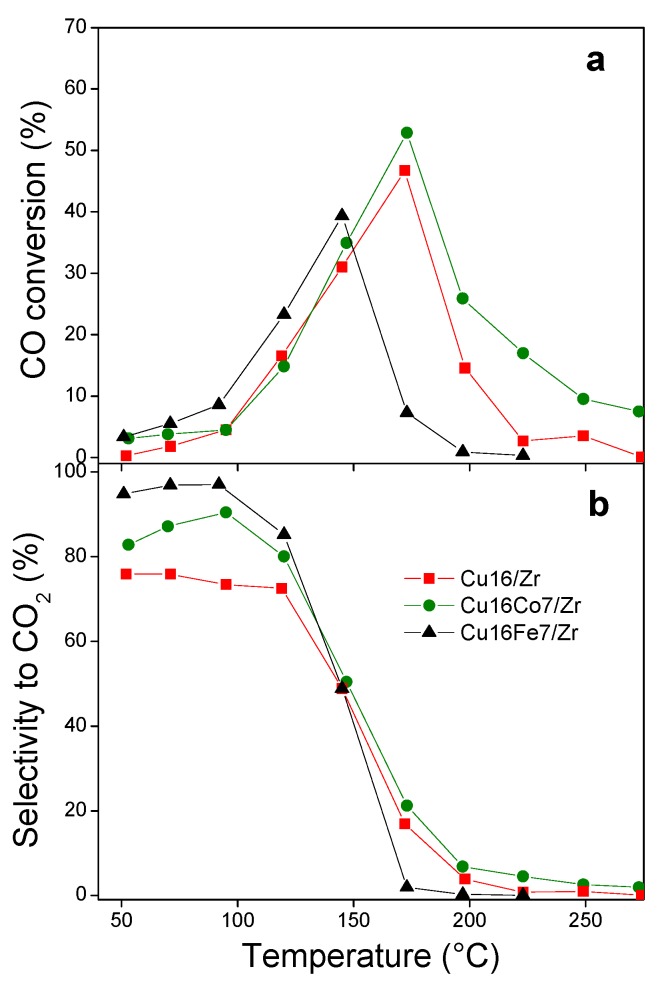
Catalytic evaluations in the preferential CO oxidation (COProx): (**a**) CO conversion; (**b**) selectivity toward CO_2_.

Given the high performance of the Cu_16_/Zr, Cu_16_Co_7_/Zr and Cu_16_Fe_7_/Zr solids, they were analyzed in the COProx reaction. The conversion curves obtained are presented in Figure 9a and show a volcano-type shape, similar to that found for catalysts based on these types of dispersed oxides in classical supports [14,36]. Initially, the conversion increase may be due to highly dispersed CuO or superficial Cu–O–Zr type sites on the zirconia [14,32], reaching a maximum of 47% (175 °C) for the Cu_16_/Zr sample. The fall in conversion at higher temperatures is probably due to a reduction in copper species dispersed in the hydrogen-rich atmosphere [14]. When comparing this behavior with that of the CO oxidation in air (Figure 6), a shift of the curves toward higher temperatures was observed for both the monometallic and bimetallic catalysts. This differs from that observed for dispersed cupric oxide crystals that exhibited a similar activity in both reactions [38], although this behavior was similar to that observed for other types of copper–iron mixed oxide catalysts [36]. This change in conversion levels may be due to structural differences between the active sites present and in the reaction mechanism operating under an oxidizing or reductive atmosphere [39]. Meanwhile, the selectivity was greater than 70% up to 120 °C, after which it fell sharply (Figure 9b).

The Cu_16_Co_7_/Zr catalyst exhibited similar characteristics to those analyzed above, with a 53% maximum conversion at the same temperature. In this sense, Co and Cu oxides would compete for the formation of M–O–Zr clusters over the zirconia support and not for the formation of Cu–Co–O–Zr species that could increase the conversion levels [14]. However, cobalt modulated the activity, given the increase in the selectivity of this system (Figure 9b). At the same time, the Cu_16_Fe_7_/Zr solid exhibited a shift of the conversion curve to lower temperature, which was compatible with the higher activity shown by this solid in COTox, previously discussed. Moreover, its selectivity was the highest of all evaluated materials (higher than 85% up to 125 °C). This behavior again accounts for the synergy between the oxide phases in this nanocatalyst.

**Figure 10:**
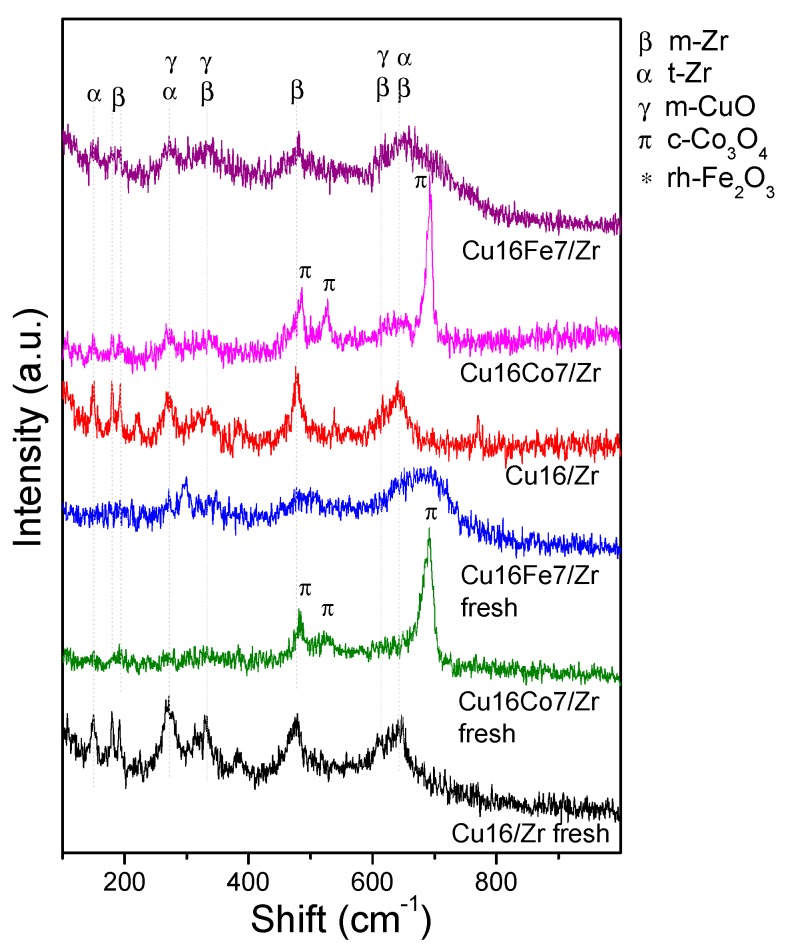
LRS spectra of the bimetallic nanocatalysts before and after the COProx reaction.

The LRS characterization of the used catalysts after the COProx evaluation is shown in Figure 10. From the analysis of the Cu_16_/Zr spectra before and after reaction, it can be inferred that the tetragonal and monoclinic phases were present in the support. Nevertheless, an increasing trend of the m-Zr strong signal at 460 cm^−1^ was observed at the expense of the t-Zr phase after reaction. This same trend can be observed in the spectra of the Co-containing materials. In the latter sample, a more acute and defined signal of Co_3_O_4_ can be additionally seen, from which the increase of the said particles under reducing atmosphere can be inferred. However, for the Cu_16_Fe_7_/Zr material, after being under reducing reaction conditions, t-Zr was still the main phase. Moreover, no signals of Cu or Fe oxides were observed, which highlights the high stability of these species in the said reaction atmosphere, which is also consistent with what was observed for this catalyst after CO oxidation in oxidizing atmosphere (Figure 6).

## 4. Conclusions

UiO-66 crystals obtained through a sustainable protocol were used as a dispersion matrix for copper, cobalt, and iron species, allowing the preparation of new nanostructured catalysts active in the oxidation of carbon monoxide. The MOF was modified with the said non-noble metals through simple and classic methods of incipient wetness impregnation, followed by controlled thermal treatments. It was shown that, by precisely tuning the treatment atmosphere (He), temperature (225 °C), and time (2h), the solid Cu/UiO-66 could be obtained having 10 wt.% copper species in a very high dispersion inside UiO-66 crystals, maintaining the structure of the MOF. This solid proved to be an active catalyst for the CO oxidation in air streams, representing a novel nanocatalyst not only for this reaction but also for others that demand a high dispersion of active copper species. It was also shown that, if the thermal decomposition treatments of the impregnated metal precursors were carried out in air at temperatures higher than 400 °C, the capacity of the MOF to host metallic species could be used to obtain non-noble metal-based catalysts supported on nano-zirconia derived from UiO-66. These controlled treatments in an air atmosphere of Cu, Co, or Fe-impregnated UiO-66 promoted the rapid development of a solid composed of tetragonal (t-Zr) and monoclinic (m-Zr) zirconia, supporting highly dispersed transition metal oxides. The derived bimetallic Cu–Fe/ZrO_2_ nanocatalyst exhibited the best levels of activity and stability both in the oxidation of CO in air and in the COProx reaction, due to synergic effects of the very close contact between such oxides in the homogeneous nanomaterial.

This study shows the potential of UiO-66 as a dispersion matrix for low-cost and abundant metals such as copper, cobalt, and iron to obtain new sustainable nanocatalysts active in the CO oxidation reaction.

## Figures and Tables

**Table 1:** Maximum decomposition temperatures (T^max^) of the fresh metal–organic framework (MOF) and of the MOF impregnated with copper (Cu/M) and that of the respective metal precursors (obtained from the derivative thermogravimetric analysis (dTGA) data).

	T^max^ N_2_^1^	T^max^ Air ^2^
Cu(NO_3_)_2_∙3H_2_O	259	255
UiO-66	555	520
Cu_5_/UiO-66	490	346
Cu_10_/UiO-66	460	318

^1^ Decomposition temperature (°C) of the maximum in the N_2_ dTGA profile. ^2^ Decomposition temperature (°C) of the maximum in the air dTGA profile.

**Table 2:** Maximum decomposition temperatures (T^max^) of the MOF impregnated with cobalt (Co/UiO-66) and iron (Fe/UiO-66), and that of the respective metal precursors (obtained from the dTGA data in air).

	T^max^ Air ^1^
Co(NO_3_)_2_∙6H_2_O	263
Fe(NO_3_)_3_∙9H_2_O	163
Co_10_/UiO-66	465
Fe_10_/UiO-66	435

^1^ Decomposition temperature (°C) of the maximum in the air dTGA profile.

## Supplementary Materials

The following are available online at https://www.mdpi.com/2079-4991/10/1/165/s1: S1: Incipient wet impregnation procedure; Figure S1: Diffractogram of the synthesized UiO-66 crystals and the pattern simulated from its crystallographic archive (CCDC 733458). The diffractogram of the benzenedicarboxylic acid ligand (BDC) is also included; Figure S2: SDTA of UiO-66(M), Cu/M, and copper precursor: (a) under N_2_ atmosphere; (b) under air atmosphere; Figure S3: Thermal stability of Co/M, Fe/M solids, and their respective precursors in air: (a) TGA; (b) dTGA; (c) SDTA. Figure S4: Co/Zr and Fe/Zr nanocatalysts: (a) catalytic behavior; (b) XRD patterns after reaction.
