# Supplementary material for: Metal–Organic Framework-Based Sustainable Nanocatalysts for CO Oxidation
Source: Nanomaterials (Basel). 2020 Jan 17;10(1):165. doi: 10.3390/nano10010165 (PMC7023304; doi:10.3390/nano10010165)
Supplement: Supplementary file 1 [file nanomaterials-10-00165-s001.pdf]

nanomaterials

Supplementary Materials

Metal–Organic Framework-Based Sustainable Nanocatalysts for CO Oxidation

Luis A. Lozano, Betina M. C. Faroldi, María A. Ulla and Juan M. Zamaro *

Instituto de Investigaciones en Catálisis y Petroquímica, INCAPE (FIQ, UNL, CONICET), Santiago del Estero 2829 (3000), Santa Fe, Argentina; llozano@fiq.unl.edu.ar (L.A.L.); bfaroldi@gmail.com (B.M.C.F.); mulla@fiq.unl.edu.ar (M.A.U.)

* Correspondence: zamaro@fiq.unl.edu.ar

nanomaterials

S1. Incipient wet impregnation procedure

The precursor solution was absorbed inside the MOF pores by capillary action. First, 0.2 g of the evacuated Zr-MOF were taken (120 °C, 15 h) and the volume of incipient wetness with water was determined. Then, an aqueous solution was prepared with the incipient wetness volume and the precursor mass required for the desired metallic loading. Afterwards, the precursor solution was added dropwise to the evacuated MOF, homogenizing with a spatula. Then, the impregnated MOF was dried at room temperature overnight and later on stove at 95 °C for 15 h. Finally, the aggregates were dispersed with an agate mortar and a fine powder was thus obtained which was stored until its use, protected from humidity and light.

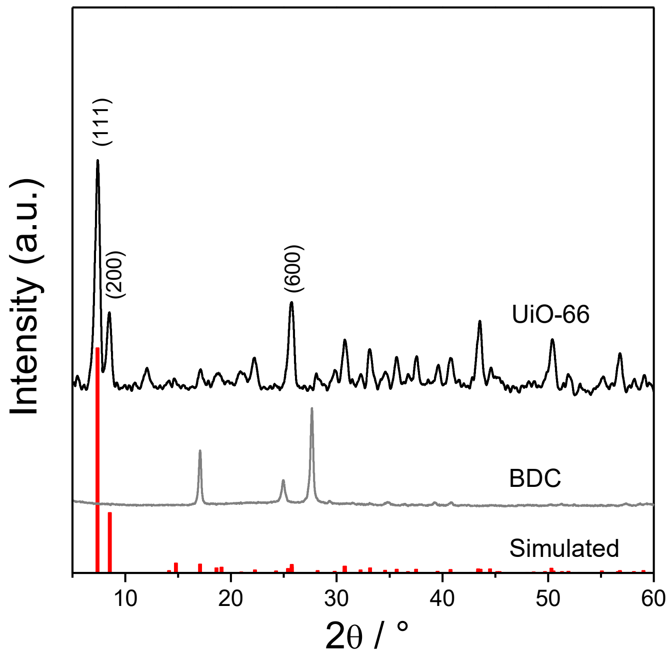

Figure S1. Diffractograms of synthesized UiO-66 and simulated from its crystallographic archive (CCDC 733458). The diffractogram of the benzenedicarboxylic acid ligand (BDC) is also included.

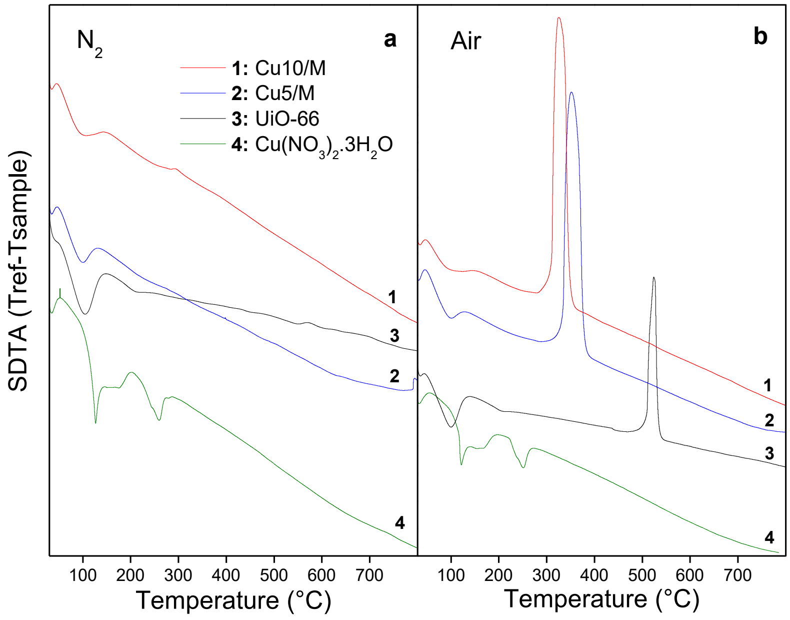

Figure S2. SDTA of UiO-66(M), Cu/M and copper precursor: (**a**) under N_2 atmosphere; (**b**) under air atmosphere.

nanomaterials

MDPI

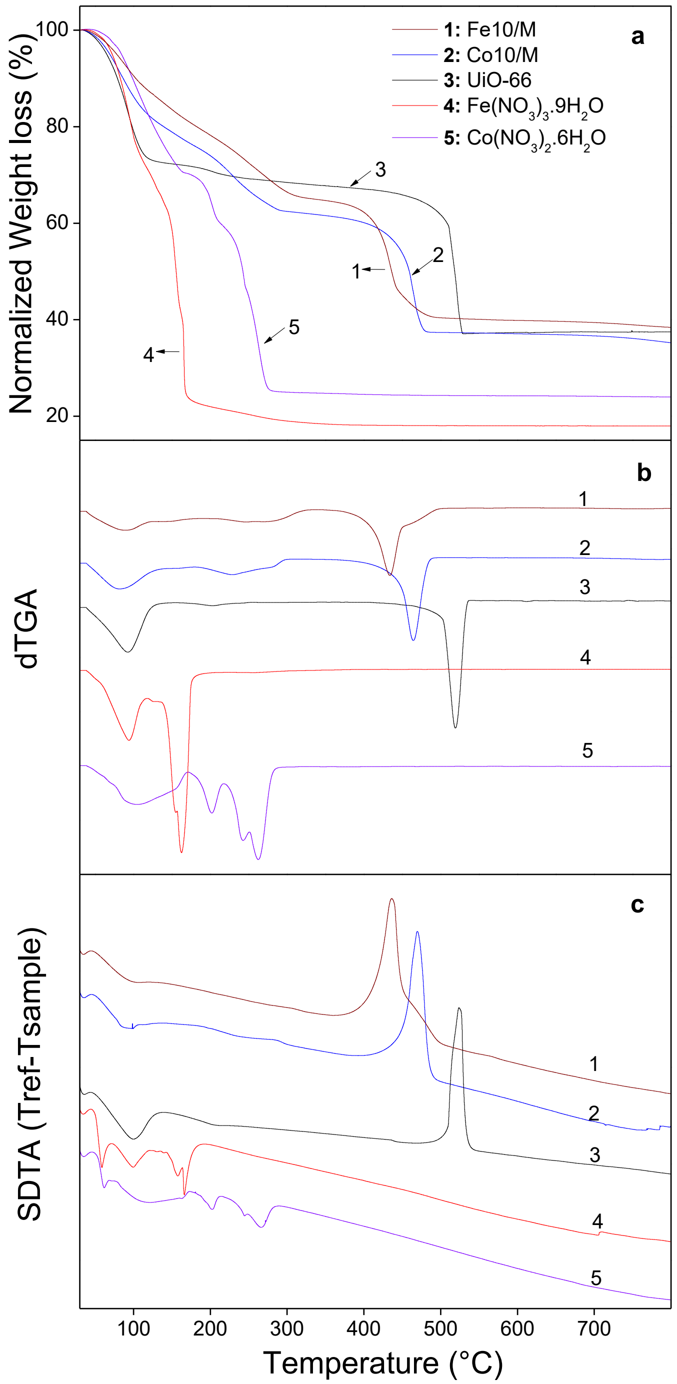

Figure S3. Thermal stability of Co/M, Fe/M solids and their respective precursors in an air: (**a**) TGA; (**b**) dTGA; (**c**) SDTA.

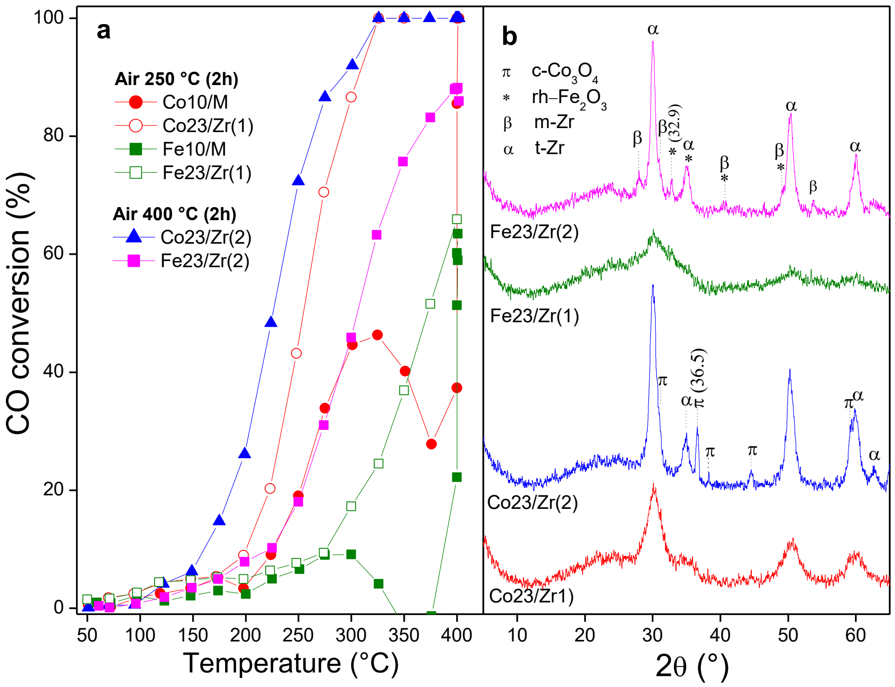

Figure S4. Co/Zr and Fe/Zr nanocatalysts: (**a**) Catalytic behavior; (**b**) XRD patterns after reaction.
